# Obstetrics, Gynecology and Pelvic Surgery

**Published:** 1896-12

**Authors:** William Warren Potter

**Affiliations:** Buffalo, N. Y. Examiner in obstetrics, New York State Medical Examining and Licensing Board


### OBSTETRICS, GYNECOLOGY AND PELVIC SURGERY.

Conducted by WILLIAM WARREN POTTER, M. D., Buffalo, N. Y.
Examiner in obstetrics, New York State Medical Examining and Licensing Board.

### PUERPERAL INSANITY.

FROM an analysis of eighty-eight cases (*Am. Jour. Insanity*, July, 1896,) and a general discussion of the subject, Pianetta, *Annali di Nevrologia*, XIII., Fasc. III., vi., concludes as follows :

1. That the so-called puerperal insanity, and especially that type developed during pregnancy, is rare.

2. That its occurrence cannot be etiologically attributed to any specific influence of the puerperal conditions, either pregnancy, the puerperal state properly speaking, or lactation, but that these are rather to be considered as occasional causes of the disorder.

3. That the mental disorder developing during the puerperal state has not special characters sufficient to separate it from other forms of insanity independent of the puerperium, either in its clinical manifestations or its course and termination, and it frequently appears under the forms of maniacal and stuporous confusional insanity.

4. That its prognosis is generally favorable and its treatment should be that of mental diseases in general, and especially be based on the etiological data and the special form in which the disorder appears in each case.

---

### TREATMENT OF PUERPERAL ECLAMPSIA.

Theophilus Parvin, of Philadelphia, (*Medical Record*,) said that puerperal eclampsia is in almost all cases caused by toxemia. Those cases in which the disease is apparently reflex in origin may be explained as resulting from an increased nervous excitability, consequent upon blood changes, and without such changes irritations would not cause convulsions. The bladder filled with urine, the loaded bowel, pain are not the essential causes ; they are only exciting causes. The spark does not cause an explosion if there be no gunpowder.

He regarded that treatment as best which acts upon the essential cause and at the same time meets symptomatic indications.

He believed that veratrum viride is better than any other drug yet employed, and that it does affect the essential cause, as well as to a great degree the symptomatic conditions, for it notably reduces the frequency of the pulse, and convulsions occur only in very exceptional cases, if the pulse be kept at sixty or less ; it increases the activity of the skin ; it reduces the temperature ; and it causes increased secretion of urine. Finally, the recovery of so large a number of mothers, about 92 per cent., when veratrum viride is employed, proves the value of the remedy.

---

### ALBUMINURIA IN PREGNANT AND PUERPERAL WOMEN.

Eklund (*Am. Jour. Obstet* , October, 1896,) writes on this subject, terminating his article with the following conclusions :

1. In handbooks for midwives, attention should be called to the necessity of examining the urine of every pregnant woman.

In case the urine is found to contain albumin, the midwife should be competent to order hot tub baths, flannel underwear, rest in the recumbent posture, mild diuretics and laxatives, beef tea with parsley, selser water with boiling milk, milk food, boiled fruit weak coffee, tea and chocolate, compound licorice powder, etc. If this hygienic treatment does not within a certain time, say a month, cause the disappearance of the albumin, a physician should be called.

2. A matter of great importance is that the pregnant woman should learn to procure for herself daily evacuation of the bowels, especially toward the end of pregnancy and in beginning labor. For this purpose dietic means should be employed chiefly, but in case of failure mild aperients should be used, such as cascara, senna, frangula, compound licorice powder, and enemata of water and salt.

3. Of the very highest importance during pregnancy, and especially during the puerperal state, is the care of the kidneys, the avoidance of all that would tend to increase the functional activity of these organs, the maintenance of equilibrium and the proper division of labor between the skin, the digestive apparatus and kidneys. If any organ can bear a greater exercise of function, it is the skin, and next in order of intolerance the intestinal tract; the lungs are far more sensitive, but the kidneys most of all.

4. No puerperal woman should be permitted to leave her bed until the urine is free from albumin, if possible.

---

### CANCER OF PREGNANT UTERUS.

Cordier, at the recent meeting of the Western Surgical and Gynecological Society, (*Am. Jour. Surg. and Gynec.*,) made an earnest plea for the removal of cancer of the pregnant uterus, and the sooner the better. Statistics show, he claims, that 80 per cent. of the children die in these cases, and 60 per cent. of the mothers, either before the end of gestation, or before getting up from the bed, so that the mortality is exceedingly high for both. These malignant troubles spread very rapidly, and if they are not operated on before confinement, they will have gone beyond operative procedure; hence he urges early removal. The doctor reported a case in which he removed the pregnant uterus at third month for cancer of cervix. Patient made a rapid recovery, and is probably cured. He believes that neither mother nor child would have been alive at the end of the period of gestation—so one life has been saved instead of two lost, a great argument in favor of radical rather than do-nothing plan of treatment.

---

### RUPTURED TUBAL PREGNANCY.

A. H. Cordier, Professor of Abdominal Surgery in the Kansas City Medical College, speaking of the necessity for operation in ruptured tubal pregnancy, (*Am. Jour. Surg. and Gynec.*,) says the abdomen should be opened in every case just as soon as the diagnosis is certain or even probable—there should be no exception to the rule. He favors flushing the belly to clean out the clots, for if done properly it does not take long, and the hot salt solution restores the lost blood serum to a considerable degree, and the heat combats shock. In 126 cases in the practice of one operator all were irrigated, and only five died. The dangers to be feared from leaving the case unoperated are: (1) Secondary hemorrhage. (2) Survival of the fetus. (3) Abscess formation. (4) Sepsis. The surgeon should not refuse to operate, even if there has been terrible loss of blood. If the pulse can still be felt at the carotids, the abdomen should be opened and bleeding checked regardless of the amount of shock present.

---

### DIFFUSE PELVIC INFLAMMATION.

Dunning, Professor of Diseases of Women in the Medical College of Indiana, (*Indiana Medical Journal,*) concludes an article on this subject as follows:

What shall we do for patients in whom the inflammation has spent its force, yet has left them with displaced and adherent uteri and appendages? They suffer more or less pain, are sterile, cannot endure wifely relations, and all are invalids in a lower or higher degree. Four plans of treatment are in vogue, and should, I think, be employed in the order mentioned: (1) The attempt to gradually loosen and replace the organs by gentle yet firm manipulation and tamponading. (2) Massage by the Brandt method. (3) Under anesthesia, the employment of Shultz's method of overcoming adhesions and restoration of the organs to the normal position. (4) And lastly, in case of failure of preceding methods, the employment of abdominal section, breaking up of adhesions, removal of the appendages, if markedly diseased, and ventrofixation or shortening of the round or broad ligaments, if retroversion exists. In all cases where it exists endometritis should be cured, and in mild cases, attended by marked pain without suppuration, galvanism will be found of value,

### PERINEAL LACERATION.

According to the Philadelphia Polyclinic, (*Med. Standard,*) in quite a number of cases laceration of the vagina and perineum may be avoided by a little patience on the part of the obstetrician, and judicious use of chloroform. This is particularly the case in primiparæ. The fetal head covered, and generally preceded by the bag of waters, makes an extremely good dilator, and ample time should be allowed for its passage through the soft parts. The patient can be easily controlled by having her lie on her side with her back toward the physician, while he makes gentle manipulation of the perineum in order to increase its distensibility. So long as the patient is making ordinary bearing-down efforts and the head is felt to advance slowly with each pain and recede slightly or remain quiescent in the intermission, no anesthetic is required ; but if the head continues steadily to advance without rest, or if, after a short quick advance, the patient makes a great outcry of pain, chloroform or ether should be given. The anesthetic has a double effect—of stopping the advancing presenting part and giving the soft parts time to dilate. Chloroform has a more relaxing effect than ether on the tissues of the vagina and perineum and is less of a uterine stimulant. It is also more easily controlled and by it the pain-sense is diminished to a greater degree without the production of unconsciousness.

### GONORRHEA IN WOMEN.

Marcus Rosenwasser states (*Cleveland Med. Jour.*, also reported in *Med. Standard,*) that while the gonococcus vegetates best within epithelial tissues, provided they are delicate, soft and moist, it also proliferates within and on the peritoneum and induces inflammation in connective tissue. Dry, horny layers of epithelium form an impenetrable barrier. It, therefore, flourishes in the vagina of young children and aged and pregnant women, but is rare in the same tissue of healthy adults. It thrives in the conjunctiva and mouth of the new-born and in the rectum of both sexes. Its presence in the urethra, the ducts of the gland of Bartholin, the vagina, the cervical canal, the uterine cavity, the tubes and on the surface of the ovaries causes inflammation in these organs. Its penetration into the lymph spaces produces broad-ligament infiltration, often leading to abscess. It can by the same route reach the hilum of the ovary and cause ovarian abscess without having passed through the tube. It can enter the circulation direct and reach remote organs, thus causing gonorrheal metastasia. The locations of choice are the urethra and the vaginal portion of the cervix. A person may in time become accustomed to his own brood of germs so that they may cease giving trouble. Let them be trans planted to new soil, they at once affect the recipient with pristine vigor. Now, if these regenerated germs be returned to their original owner, they will initiate as vicious a recurrence as though they had never been there before. This is an explanation of "latent" and recurrent gonorrhea. Thus, too, a man and his wife may finally become indifferent to their own germs, but a third party may be infected by either. This third party may in turn reinfect husband or wife, and these again one another. Chronic gonorrhea, therefore, affords no immunity against an acute attack. Infection may be propagated by other means than sexual congress in its natural or perverted forms. Towels, sheets, sponges, baths taken in common, infected instruments, contact with secretion at birth, all are known to have been carriers of infection. In connection with infection at birth, by passage through a gonorrheic vagina, it should be remembered that Marx has often observed (*Brit. Med. Jour.*) symptoms precisely resembling those indicating acute inflammation of the appendages in the adult. A child subject to vulvitis, yet otherwise in good health, is suddenly seized with fever and nausea ; pain is felt in the hypogastrium radiating along one or both thighs. Frequent desire to micturate and pricking feelings when urine is passing are often felt. Rectal exploration with the little finger shows characteristic deposits on each side of the uterus. Marx finds this physical symptom common and believes that the tubes are generally involved in chronic vulvitis In such a case the pain at puberty is very violent, and masturbation may light up old-standing inflammatory troubles. Old lesions of this kind are certainly liable to become acute in young recently-married women through excessive coitus. It is quite a mistake to accuse the husband or to suspect that the wife has recently suffered from specific discharge in many such cases. The focus of disease has often dated from early childhood. It should be remembered that, as Dr. J. V. Shoemaker remarks (*Med. Bull.*):

Gonorrhea in the female occupies a different anatomical site than in the male, being, in a large majority of cases, a vaginitis. The infecting fluid is usually deposited in the upper part of this canal, and excites inflammation of the mucous membrane in that situation. The disease generally originates from subacute, and particularly chronic, cases in the male partners, as in the acute state erections are painful and men are not in a state of mind or body which disposes them to seek the society of women. Urethritis in women is more commonly associated with gonorrheal inflammation of the vulvar or lower portions of the vagina. The diagnosis of gonorrhea is seldom difficult. The discharge is much more abundant in vaginitis than in leucorrhea. An unusually copious leucorrheal flow might stimulate the declining stage of a gonorrhea, but the use of the speculum will enable us to distinguish between the two affections.

